# Supplementary material for: Drug reformulation for a neglected disease. The NANOHAT project to develop a safer more effective sleeping sickness drug
Source: PLoS Negl Trop Dis. 2021 Apr 15;15(4):e0009276. doi: 10.1371/journal.pntd.0009276 (PMC8078842; doi:10.1371/journal.pntd.0009276)
Supplement: S1 File — Fig A—Pentamidine is returned to the blood from the capillary endothelial cell by P-gp and MRP. Pluronic P85 inhibits-mediated efflux (e.g. P-gp and MRP transport) by two mechanisms: the first through membrane fluidisation and the second through transient ATP depletion. These effects are believed to be mediated by unimers (single polymer chains) [22, 20]. Inhibition of efflux should facilitate the accumulation of pentamidine in the human cerebral capillary endothelium and the murine choroid plexus epithelium, leading to higher concentrations of pentamidine. Fig B—Pyrene fluorescence intensity dependence on pluronic concentration for F68, P85 and P105. The CMC was determined using 18 different concentrations (range 0.0001 to 1 w/v%) of pure P85, P105 and F68. The value at each concentration is the mean of two samples, each prepared from a separate preparation of the stock solution. As expected, the curves show two inflection points. The first was taken as the CMC. Fig C—Typical partition data for PTI fluorescence as a function of F68 and P105 concentration. Fig D—Drug release from dialysis cells measured over time. The experiments were conducted in water at 37°C for concentrations as close as possible to in vitro conditions, within experimental limitations, namely, 1% w/v of Pluronics and 10mM PTI. No significant differences between the Pluronics were observed and drug release is diffusion controlled (Fickian diffusion) under the experimental conditions. Pluronics micelles are not a barrier to drug release. Fig E—SANS Pluronic data at 37°C. A) P85 5% B) F68 5% C) P85 5% / PTI 1% D) F68 5% / PTI 1% E) P85 5% / PTI 3% F) F68 5% / PTI 3%. Fig F—The average number of Pluronic molecules found in a micelle (Nagg) and the number of micelles in our system (after they have equilibrated) (Nmic) as a function of the concentration of the F68 Pluronic in a system that contains F68 and 0.01 w/v% of L61 Pluronic. In both plots, the black curve represents the results when considerin [file pntd.0009276.s001.docx]

**Full title:**

Drug reformulation for a neglected disease.

The NANOHAT project to develop a safer more effective sleeping sickness drug.

**Short title:**

Pentamidine reformulation to improve safety and efficacy against Human African Trypanosomiasis

Lisa Sanderson^1¶^, Marcelo da Silva^1¶^, Gayathri N. Sekhar^1^, Rachel C. Brown^1^, Hollie Burrell-Saward^2^, Mehmet Fidanboylu^1^, Bo Liu^3^, Lea Ann Dailey^1#^, Cécile A. Dreiss^1^, Chris Lorenz^4^, Mark Christie^1^, Shanta J. Persaud^3^, Vanessa Yardley^2^, Simon L Croft^2^, Margarita Valero^5^ and Sarah A. Thomas^1^*

^1^King’s College London, Institute of Pharmaceutical Science, Franklin-Wilkins Building, Stamford Street, London, United Kingdom

^2^Faculty of Infectious and Tropical Diseases, London School of Hygiene and Tropical Medicine, London, United Kingdom

^3^King's College London, Department of Diabetes, School of Life Course Sciences, Faculty of Life Sciences & Medicine, London, United Kingdom

^4^King's College London, Theory & Simulation of Condensed Matter Group, Department of Physics, Strand, London, United Kingdom.

^5^Physical Chemistry Department, Faculty of Pharmacy, University of Salamanca, Salamanca, Spain

^#^ **Current address**: Dept. of Pharmaceutical Technology and Biopharmacy, University of Vienna, Vienna, Austria

^¶^equal contributors

*Corresponding author: sarah.thomas@kcl.ac.uk

**Text A in S1 File. Purity of excipients**

All Pluronic formulations (F68, P105, P85 and L61) were tested for possible contamination due to the synthesis process and met specifications established by the US Pharmacopeia convention NF32 monograph for polaxamers [1], in that the EO, PO and p-dioxane in the Pluronic were below 1, 5 and 5 parts per million (ppm), respectively. This analysis was performed by an external specialist laboratory (Butterworth laboratories, Teddington, UK).

**Text B in S1 File.** The partition coefficient was obtained following the procedure described by Kabanov and co-workers [2], which is described briefly below. The partition coefficient is defined as:

$P=\frac{c_{m}}{c_{bulk}}$ Eq. 1

where *c_m_* is the PTI concentration in the micelle core and *c_bulk_* is the PTI concentration in the bulk solvent.

The partition coefficient *P* can be obtained from[2]:

$P=\frac{1}{0.01*\alpha*v}$ Eq. 2

where, *v* (cm³/g), the partial molar specific volume, is defined as:

$v=\frac{1}{\rho_{0}}*\frac{1-(\rho_{s}-\rho_{0})}{C}$ Eq. 3

with ρ_0_ (g·cm^-^³) the solvent density, ρ_s_ (g·cm^-^³) the density of the solution and *C* the polymer concentration (g·mL^-1^).

α is defined as the angular coefficient of the plot ($\frac{I- I_{0}}{I_{max}-I_{0}}$)^-1^ vs. (Pluronic concentration – cac)^-1^, where *I* is the fluorescence intensity of the solution, *I_0_* is the intensity with no Pluronic present and *I_max_* is the intensity at saturating concentration of the Pluronic, cac the critical aggregation concentration.

**Text C in S1 File.** Small-angle neutron scattering experiments were performed on the LOQ instrument. LOQ uses incident wavelengths from 2.2 to 10.0 Å, sorted by time-of-flight, with a fixed sample-detector distance of 4.1 m, which provided a range of scattering vectors (*q*) from 0.009 to 0.29 Å^-1^.

The samples used in the SANS experiments were prepared in D_2_O to optimise the contrast with the protonated polymer. The samples were placed in clean disc-shaped quartz cells (Hellma) of 1 and 2 mm path length and the measurements were carried out at 25°C and 37°C. All scattering data were first normalised for sample transmission and then background-corrected using a quartz cell filled with D_2_O (this process also removes the inherent instrumental background arising from vacuum and windows) and finally corrected for the linearity and efficiency of the detector response using instrument-specific software package. The data were then converted to the differential scattering cross-sections (in absolute units of cm^-1^) using the standard procedures at ISIS [3,4].

The curves were fitted to a core-shell sphere model combined with a structure factor for hard spheres, implemented in the Sasview software (SasView, http://www.sasview.org accessed 23 November 2014). The initial fitting assumptions were a dry PPO core, i.e., the scattering length density (SLD) for the core was set to the SLD of PPO (3.44×10^-7^ Å^-2^), the solvent as D_2_O (SLD of 6.38×10^-6^ Å^-2^) and the shell as dry PEO (SLD 6.4×10^-7^ Å^-2^). The SLD of core and solvent were kept fixed while the shell was left to vary as a high level of hydration of PEO is expected, as a result, the returned SLD should be an intermediate value between D_2_O and PEO. After obtaining a stable set of parameters, the SLD of the core was also let free to vary. Input values for the core radius and shell thickness were based on hydrodynamic radius values obtained by DLS. A term to compensate for polydispersity was included for Pluronic (around 0.20), as well as a structure factor (*S*(*q*)), corresponding to a hard sphere model, to account for intermicellar interactions.

Assuming that the shell SLD is a linear combination of the EO SLD and D_2_O SLD, i.e., the densities are additive, it is possible to calculate the solvent volume fraction in the shell using the following expression:

$\chi_{\mathrm{solv}}\text{=}\frac{{SLD}_{shell}-{SLD}_{EO}}{{SLD}_{D_{2}O}-{SLD}_{EO}}$ Eq. 5

The amount of solvent molecules in the shell than can be obtained from the ratios of the shell volume by the solvent molecular volume weighted by the solvent volume fraction:

$n_{D_{2}O}=\frac{\chi_{solv}.V_{shell}}{V_{D_{2}O}}$ Eq. 6

Furthermore, the total micelles volume is the sum of both Pluronics and D_2_O molecular volumes weighted by their respective volume fractions. Therefore, combining the total micelle volume (shell + core) minus the solvent contribution, it is possible to obtain the amount of Pluronic molecules present in the micelle and calculate the aggregation number of the micelles.

$N_{agg}=\frac{V_{micelle}-\chi_{solv}V_{shell}}{V_{pluronic}}$ Eq. 7

The molecular volume of the Pluronic was approximated from the Pluronic molar volume (*v_pluronic_*) in water.

$V_{pluronic}=\frac{v_{pluronic}}{N_{AV}}$ Eq. 8

**Text D in S1 File.**

**Chemicals.** Cyanmethaemoglobin (CMH) reagent, haemoglobin standard, Ca^2+^/Mg^2+^ free Dulbecco's Phosphate Buffered Saline (DPBS), DMSO, and Triton X-100 were purchased from Sigma-Aldrich, Dorset, UK.

**Research Donor Blood.** Blood samples from two healthy volunteers were drawn under the guidelines of the Research Ethics Committee South East London REC 4 (10/H0807/99). Blood was collected in BD vacutainer tubes containing lithium heparin as anticoagulant.

**Haemolysis Assay.** The assay is based on the protocol detailed by [6]. To determine the total blood haemoglobin, the CMH method was used to map a standard curve based on the absorption wavelength at 540 nm. Nine calibration standards were produced by preparing a stock solution of 5 mg/ml (standard 1) using haemoglobin standards in CMH reagent and serially diluting it to produce further standards of concentrations 2.5, 1, 0.80, 0.40, 0.20, 0.10, 0.05, and 0.025 mg/ml. 10% Triton X-100 (v/v) was prepared using distilled water to be used as the positive control. A 0.05% solution of DMSO was prepared using distilled water and used as one of the negative controls. 0.9% saline was another negative control. Pluronic samples were prepared in 0.9% saline. The test concentrations of Pluronic (P85, P105 or F68) used were 0.01%, 0.1%, 0.25%, 1% or 5%.

A 5 ml vial of whole blood was centrifuged at 800 g for 15 minutes at room temperature. The supernatant was removed, and the remainder was used to determine the plasma free haemoglobin (PFH) concentration.

200 μl x 2 of each of the calibration standards prepared were then transferred onto the 96-well plate and 200 μl x 4 of CMH reagent (control 1). Total blood haemoglobin (TBH) was then prepared by adding 20 μl of the whole blood with 5 ml of CMH reagent. 200 μl x 6 of TBH was transferred to the plate. 6 other wells were filled with 100 μl of plasma to which 100 μl of CMH reagent was added. After shaking it gently for a few minutes, the absorbance was read at 540 nm. Once the total haemoglobin concentration was adjusted to 10 mg/ml using Ca^2+^/Mg^2+^ free DPBS, 20 μl x 3 of the blank (control 2 0.9% saline), positive control, negative control, or Pluronic samples were added to Eppendorf tubes. 160 μl of Ca^2+^/Mg^2+^ free DPBS and 20 μl of whole blood were then added to each tube except for one. 20 μl of Ca^2+^/Mg^2+^ free DPBS was added instead. These served as control 3 without any blood enabling us to determine any interactions from Pluronic with the assay. These tubes were incubated for 3 hours ± 15 minutes in a temperature-controlled shaker (THERMOstar, BMG labtech, Offenburg, Germany) at 37 °C and 120 rpm. After the incubation, these were centrifuged at 800 × g for 15 minutes at room temperature. 100 μl of the test samples and the controls were transferred to the 96-well plate. 100 μl of CMH reagent was added to all these before measuring the absorbance spectrophotometrically at 540 nm. Using the calibration curve mapped earlier, the haemoglobin concentration in each of the wells was determined. The dilution factor of 18 was also considered when calculating the haemoglobin concentration. Haemoglobin concentration was converted to percentage haemolysis compared to the negative control 0.05% DMSO. A significant increase compared to control at the 5% level was taken as positively haemolytic.

**Text E in S1 File.** **Sensitivity of MDCK-MDR cells to Pluronic**

Permeability assay with 5 μCi (0.9 μM) [^14^C(U)]sucrose alone in the presence of varying concentrations of Pluronic was used to assess the effect of Pluronic on monolayer integrity. Sucrose is a paracellular permeability marker and therefore any effects of Pluronic will lead to increased diffusion of [^14^C(U)]sucrose from apical to basolateral chambers because of compromised BBB integrity. The assay was carried out for an hour in the apical to basolateral direction only and the Papp of radiolabelled sucrose determined at the end of the assay. Control for the assay was HBSS buffer alone.

**Text F in S1 File. UPLC MS/MS**

Quantification of pentamidine isethionate was carried out on an ABSciex API5500 QTrap triple quadrupole mass spectrometer coupled to an Agilent 1290 Infinity UPLC system. A 10μl sample from the apical or basolateral chamber was diluted with 80 μl buffer and 160 μl methanol and centrifuged for 20 minutes at 1,400 × *g*. After centrifugation 90 µl of this was removed and diluted with 10 µl of the internal deuterated standard to give a final concentration of 10 nM. This was injected onto an Acquity™ HSS T3 (1.8 µm) column 2.1 x 50 mm (Waters Ltd, Herts, UK), equilibrated at 70 °C. Separation was carried out by gradient elution using a mixture of 0.1% formic acid in H_2_O (solvent A) and 0.1% formic acid in acetonitrile (solvent B), at a flow rate of 600 µL/min. Upon injection (10 μl), the mobile phase was held at initial conditions of 100% solvent A for 0.05 minutes, the concentration of solvent B was then increased to 95% at 1.00 minute post-injection using a linear gradient and held for a further 0.40 minutes. The mobile phase was then returned to 100% solvent A at 1.41 minutes and held for a further 0.39 minutes. The eluent was analysed by MS/MS under positive ion electrospray mode and the multiple reaction monitoring transitions for pentamidine and deuterated pentamidine were 341.174 to 119.984 m/z and 345.212 to 120.028 m/z, respectively. Calibration curves were prepared using pentamidine and the internal standard over a concentration range of 0.0078 to 12 µM. The column was washed with a weak wash: 9:1 H_2_O: acetonitrile and strong wash: 4:3:3 methanol: isopropyl alcohol: acetone + 1% acetic acid between samples.

**Text G in S1 File**. The capillary depletion method uses dextran density gradient centrifugation to produce a vascular endothelial cell-enriched pellet and a brain parenchyma-containing supernatant from homogenized brain tissue. Thus the partitioning of a drug between the endothelial cells and the post-vascular brain parenchyma can be examined [7]. Briefly, the brain tissue was weighed and homogenized in a Dounce homogenizer with 3 ml/g capillary depletion buffer [7] and 4 ml/g 26% dextran. The homogenate was separated by centrifugation at 5,400g and 4°C for 15 minutes. Homogenate, pellet and supernatant samples were solubilized and counted for radioactivity using the method described above.

**Text H in S1 File. Haemolysis assay**

All the Pluronics (P85, F68 and P105) at each of the tested concentrations (0.01%, 0.1%, 0.25%, 1% or 5%) and both the negative controls (0.05% DMSO or 0.9% saline) did not cause any haemolysis (0%) of the human cells. In contrast the positive control, 10% Triton X-100, caused haemolysis (***p<0.001 compared to the negative controls).

**Text I in S1 File. Permeability assay (MDCK-MDR) to assess membrane integrity**

[^14^C(U)]sucrose is an inert, polar molecule which normally does not cross cell membranes, but may cross between cells through the paracellular cleft. Significant differences in [^14^C(U)]sucrose Papp values existed in the presence of all tested concentrations of P85, and 0.5% and 0.01% P105 compared to [^14^C(U)]sucrose Papp in the absence of Pluronic (Fig S7) indicating loss of monolayer integrity. No tested concentration of F68 significantly affected the radiolabelled sucrose Papp values.

**Text J in S1 File.** The ability of pentamidine isethionate to cross the MDR1-MDCK cell monolayers in the apical (luminal) to basolateral (abluminal) direction was limited as the concentration of pentamidine isethionate in the basolateral chamber were below the limits of UPLC-MS/MS detection, even when the pentamidine isethionate concentration was increased to 20 μM. The lower limit of quantification (LLQ) for UPLC-MS/MS method was ≤ 7.8 nM which is equivalent to <0.039% of the dose in the donor (apical) chamber. These results would suggest that pentamidine is a ‘CNS negative’ drug. The mass balance (% recovery) results suggest that up to 25% may have been trapped either in the endothelial cells or by non-specific binding to the plastic plate or the polycarbonate membrane and this may contribute to the reduced recovery. It is important to note that the two amine groups of pentamidine are ionized at physiological pH (pKa is 11.4) and are likely to react with static charges on the surface of plastics. Interestingly the presence of Pluronic increased the mass balance results by 10-20%. In contrast the movement of pentamidine isethionate (20μM) across the monolayer in the basal to apical direction was measurable, the calculated P_app_ being 0.418 x 10^-6^ cms^-1^. Taken together with the absence of a detectable movement of pentamidine isethionate in the apical to basolateral direction these data would indicate the presence of an efflux mechanism for this molecule, likely MDR1. The presence of Pluronics (F68, P105 or P85 at concentrations of 0.01%, 0.1% or 0.5%) did not affect the distribution of pentamidine isethionate across the monolayer in either direction.

MDR1-MDCK permeability assay experiments were also performed using radiolabelled pentamidine and liquid scintillation counting. The rate of transport of [^3^H(G)]pentamidine (9 nM) across MDR1-MDCK monolayer was examined and in contrast to the results achieved with the pentamidine isethionate was measurable in both directions (Table 9 in main document). In these experiments the highest concentrations of P85 (0.5%) and P105 (0.5%) affected the integrity of the cell monolayer and the Papp for [^14^C(U)]sucrose was increased to 4.8 and 4.3 x 10^-6^ cm/s, respectively. The [^14^C(U)]sucrose P_app_ for all other experiments was 1.61±0.15 x 10^-6^ cm/s. The presence of the Pluronics (F68, P105 or P85) at concentrations of 0.01% and 0.1% did not significantly increase the distribution of [^3^H(G)]pentamidine across the MDR1-MDCK monolayer measured over 60 minutes confirming the results obtained using pentamidine isethionate and the UPLC-MS/MS detection method.

**Text K in S1 File. PLURONIC P85**

Co-formulation of 15.7 nM [^3^H(G)]pentamidine with Pluronic P85 did not significantly increase [^3^H(G)]pentamidine accumulation in any of the brain regions examined (Table G in Text K).

In fact, overall there was a decrease in the [^14^C(U)]sucrose-corrected uptake of [^3^H(G)]pentamidine with P85 at 0.01% (p<0.001) and at 0.1% (p<0.01) (Two-Way ANOVA), but these decreases were not statistically significant when individual brain regions were examined using Bonferroni’s pairwise comparisons.

Table G in Text K shows the results of capillary depletion analysis of the brain tissue after 10 minutes of perfusion in the presence or absence of the Pluronic, P85. [^3^H(G)]pentamidine accumulated in the capillary endothelial cells (pellet) of control mice while less than 2% of the plasma concentration crossed the basolateral membrane to reach the parenchyma (supernatant). These results are in good agreement with our previously published data[8]. There appeared to be an overall reduction in this accumulation of [^3^H(G)]pentamidine into the endothelial cells and consequently a reduction in the parenchyma when [^3^H(G)]pentamidine was co-formulated with 0.01% and 0.1% P85, but the reduction did not attain statistical significance (Two-Way ANOVA with Bonferroni’s pairwise comparisons).

Whilst there appeared to be an overall inhibitory effect of Pluronic on the transport of [^3^H(G)]pentamidine across the BBB, there was a significant, 3-fold increase in the uptake of [^3^H(G)]pentamidine into the pituitary gland after 10 minutes of perfusion with 0.1% and 0.5% P85 (p<0.05 at both concentrations; Two-way ANOVA with Bonferroni’s pairwise comparisons). This enhanced uptake of pentamidine appeared to be associated with an approximate 2-fold increase in accumulation of [^14^C(U)]sucrose from 20.5±3.9% ([^14^C(U)]sucrose alone) to 35.0±5.5% (+0.01%P85), 43.0±4.0% (+0.1% P85) and 34.3±7.9% (+0.5% P85). A similar effect was observed in the choroid plexus sampled from a few individual mice that were perfused with pentamidine co-formulated with P85 at concentrations above the CMC. This resulted in a 2-fold increase in the mean uptake of [^3^H(G)]pentamidine which was not statistically significant. P85 did not affect accumulation of [^3^H(G)]pentamidine or [^14^C(U)]sucrose by the pineal gland.

**Text L in S1 File. PLURONIC P105**

No significant differences were observed in the uptake of either the vascular space marker [^14^C(U)]sucrose or [^3^H(G)]pentamidine into the pineal gland, choroid plexus or pituitary gland after 10 minutes of perfusion with [^3^H(G)]pentamidine co-formulated with P105 as shown in Table H in Text L (p>0.05 for each Pluronic concentration and circumventricular organ; Two-way ANOVA with Bonferroni’s pairwise comparisons).


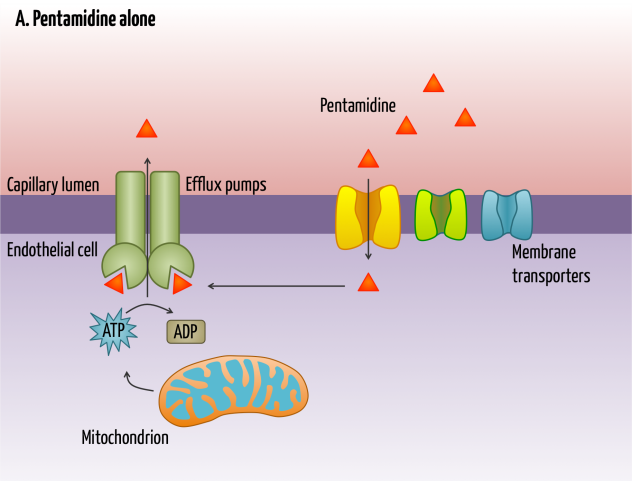

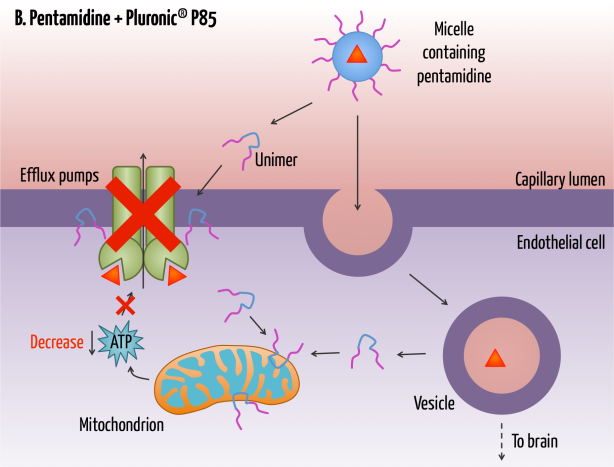


**Fig A.** **Pentamidine is returned to the blood from the capillary endothelial cell by P-gp and MRP. Pluronic P85 inhibits-mediated efflux (e.g. P-gp and MRP transport) by two mechanisms; the first through membrane fluidisation and the second through transient ATP depletion**. These effects are believed to be mediated by unimers (single polymer chains) [9][10]. Inhibition of efflux should facilitate the accumulation of pentamidine in the human cerebral capillary endothelium and the murine choroid plexus epithelium, leading to higher concentrations of pentamidine.

**Fig B**. **Pyrene fluorescence intensity dependence on pluronic concentration for F68, P85 and P105**. The CMC was determined using 18 different concentrations (range 0.0001 to 1 w/v%) of pure P85, P105 and F68. The value at each concentration is the mean of two samples, each prepared from a separate preparation of the stock solution.  As expected the curves show two inflection points. The first was taken as the CMC.

**Fig C.** **Typical partition data for PTI fluorescence as a function of F68 and P105 concentration.**

**Fig D. Drug release from dialysis cells measured over time. The experiments were conducted in water at 37°C for concentrations as close as possible to *in vitro* conditions, within experimental limitations, namely, 1% w/v of Pluronics and 10mM PTI**. No significant differences between the Pluronics were observed and drug release is diffusion controlled (Fickian diffusion) under the experimental conditions. Pluronics micelles are not a barrier to drug release.

**Fig E.** **SANS Pluronic data at 37°C.** A) P85 5% B) F68 5% C) P85 5% / PTI 1 % D) F68 5% / PTI 1 % E) P85 5% / PTI 3 % F) F68 5% / PTI 3 %.

D

E

F

B

A

C

**
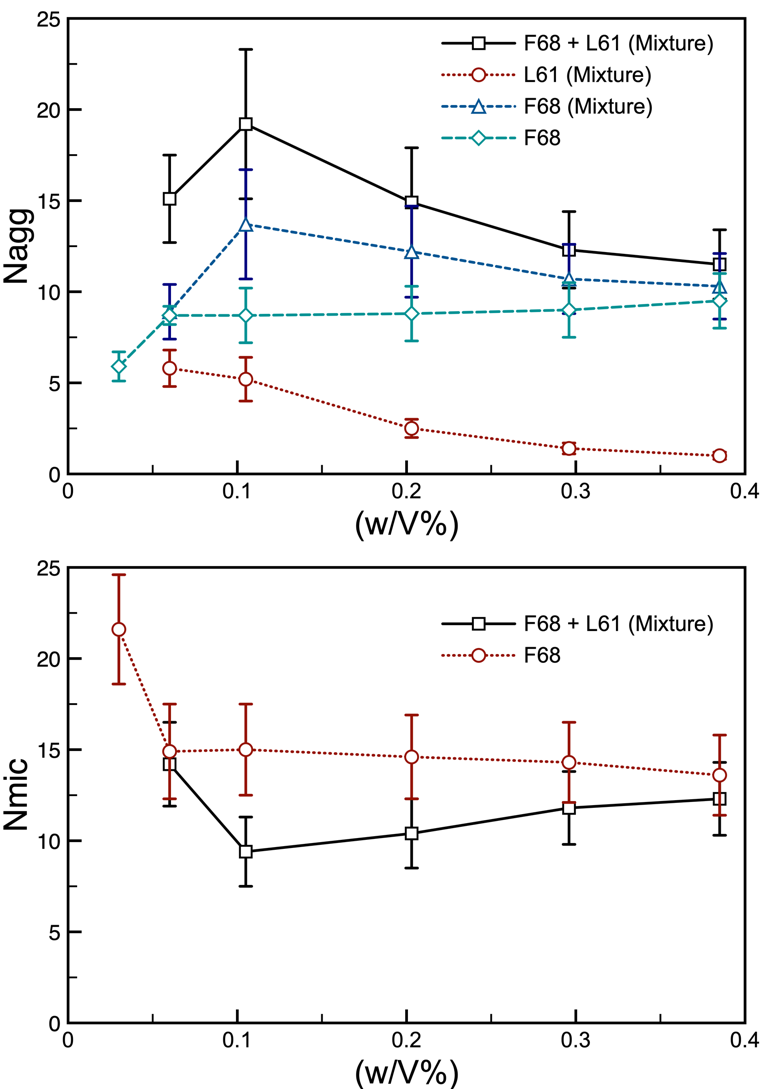
**

**Fig F.** **The average number of Pluronic molecules found in a micelle (N_agg_) and the number of micelles in our system (after they have equilibrated) (N_mic_) as a function of the concentration of the F68 Pluronic in a system that contains F68 and 0.01 w/v% of L61 Pluronic**. In the both plots, the black curve represents the results when considering both the L61 and F68 polymers in the mixture, and the blue dashed curve represents the data from the pure F68 simulated systems. In the top curve, the red curve represents the number of F68 in a micelle which contains both F68 and L61, and the green curve represents the number of L61 in a micelle. The results show that as we increase the concentration of F68, and therefore make the system more and more like the pure F68 system, the number of polymer molecules in a micelle and the number of micelles converge to that observed in the pure F68 system, as expected. Interestingly, it seems that from our simulations that L61 causes the aggregation of F68 to become slightly enhanced as the number of F68 in the average micelle is always larger than that found in the pure F68 micelles, which naturally results in their being fewer micelles.


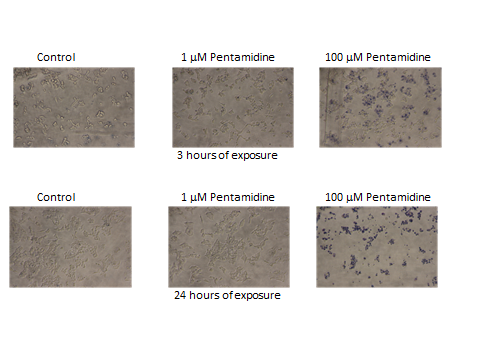


**Fig G.** **Effects of exposure of MIN6 β-cells to 0 (control), 1 or 100 μM pentamidine for 3 and 24 hours. Trypan blue uptake.** Blue staining demonstrates cells of compromised viability, highlighting the toxicity of 100 μM pentamidine to these cells after 3 hours exposure.


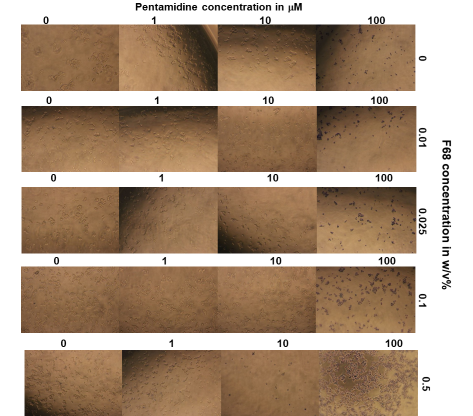


**Fig H.** **Effects of exposure of MIN6 β-cells to 0, 1, 10 or 100 μM pentamidine and 0, 0.01, 0.025, 0.1 or 0.5% w/v% F68 for 24 hours**. Trypan blue uptake. Blue staining demonstrates cells of compromised viability, highlighting the toxicity of 100 μM pentamidine and 0.5% F68 to these cells.

**Fig I.** **Apical to basolateral permeability of [^14^C]sucrose in the presence of P85, P105, and F68 concentrations measured over 60 minutes**. Significant differences compared to control (no pluronic) was observed in the presence of P85 and P105 (***p<0.001, **p<0.01). All data are expressed as mean ± S.E.M, n= 3 wells. Data were analysed using one-way ANOVA with SigmaPlot 13.0.

**Table A. Single point CNS side effect screening of pentamidine at a concentration of 1.0E^-5^ M (PERKIN ELMER study no. 13-9625).** Details of the assay, reference K_i_, reference compound and the radioligand/substrate used in the CNS side effects panel ligand binding assay are described. Values are expressed as the percent inhibition of specific binding and represent the average of duplicate tubes. Pentamidine could be described as active at that binding site if it showed inhibition of 50% or greater (see shaded boxes/compound hit true). Inhibition in the range of 20% to 49% indicated marginal activity at the receptor site and were not investigated further. The baseline range in these assays was considered -20% to +20% inhibition of binding activity. Compounds showing results in this range were considered inactive at this site. K_i_ is the inhibitory constant and is reflective of the binding affinity of the drug for the receptor.

| **Assay Type** | **Percent Inhibition** | **Assay Name** | **Reference K_i_** | **Reference Compound** | **Radioligand/Substrate** | **Compound Hit** |
| --- | --- | --- | --- | --- | --- | --- |
| NEUROTRANSMITTER RELATED | 101.03% | Imidazoline, I2 [central] | 3.81E-10 | 2-BFI | [^3^H]2-BFI | TRUE |
| NEUROTRANSMITTER RELATED | 100.77% | Adrenergic, Alpha 1, Non-selective | 1.21E-8 | Phentolamine | [^3^H]7-MeOxy-Prazosin | TRUE |
| ENZYMES | 99.99% | Oxidase, MAO-B, Peripheral | 5.86E-8 | Ro 16-6491 HCl | [^14^C]Phenylethylamine | TRUE |
| NEUROTRANSMITTER RELATED | 90.90% | Muscarinic, Non-selective, Central | 3.11E-10 | Atropine sulfate | [^3^H]QNB | TRUE |
| ENZYMES | 89.42% | Oxidase, MAO-A-Peripheral | 2.34E-8 | Tetrindole mesylate | [^14^C] - 5HT | TRUE |
| NEUROTRANSMITTER RELATED | 76.00% | Opioid, Non-selective | 1.31E-9 | Naloxone HCl | [^3^H]Naloxone | TRUE |
| NEUROTRANSMITTER RELATED | 75.89% | Dopamine Transporter | 1.26E-8 | GBR12909 | [^3^H]WIN 35,428 | TRUE |
| NEUROTRANSMITTER RELATED | 71.07% | Histamine, H2 | 1.80E-8 | Tiotidine | [^125^I]-Aminopotentidine | TRUE |
| NEUROTRANSMITTER RELATED | 61.05% | Serotonin Transporter | 4.29E-8 | Imipramine HCl | [^3^H]Citalopram, N-Methyl | TRUE |
| NEUROTRANSMITTER RELATED | 56.14% | Adrenergic, Alpha 2, Non-selective | 1.26E-8 | Phentolamine | [^3^H]RX821002 | TRUE |
| NEUROTRANSMITTER RELATED | 55.37% | Adrenergic, Beta, Non-selective | 4.55E-9 | Alprenolol HCl | [^3^H]DHA | TRUE |
| ION CHANNELS | 46.94% | Sodium, Site 2 | 2.29E-6 | Aconitine | [^3^H]Batrachotoxin A 20-a-Benzo | FALSE |
| NEUROTRANSMITTER RELATED | 36.62% | Opioid, Orphanin, ORL1 (h) | 1.83E-9 | Nociceptin | [^3^H] Nociceptin | FALSE |
| NEUROTRANSMITTER RELATED | 31.10% | GABA A, Agonist Site | 1.64E-8 | GABA | [^3^H]GABA | FALSE |
| NEUROTRANSMITTER RELATED | 27.48% | Dopamine, D1 (h) | 2.42E-10 | SCH23390 | [^3^H]-SCH23390 | FALSE |
| NEUROTRANSMITTER RELATED | 23.45% | Melatonin, Non-selective | 8.39E-10 | Melatonin | [^125^I]-2-Iodomelatonin | FALSE |
| ENZYMES | 23.21% | Esterase, Acetylcholine | 3.85E-7 | Eserine (Physostigmine) | Acetylthiocholine | FALSE |
| NEUROTRANSMITTER RELATED | 21.39% | Norepinephrine Transporter | 6.51E-10 | Desipramine HCl (DMI) | [^3^H]Nisoxetine | FALSE |
| NEUROTRANSMITTER RELATED | 20.54% | GABA A, BDZ, alpha 1 site | 7.21E-10 | Ro15-1788 (Flumazenil) | [^3^H]Flunitrazepam | FALSE |
| NEUROTRANSMITTER RELATED | 20.12% | Dopamine, D2s (h) | 2.54E-10 | Haloperidol | [^3^H]-Raclopride | FALSE |
| GROWTH FACTORS/HORMONES | 17.05% | Corticotropin Releasing Factor, Non-selective | 1.07E-8 | Tyr0-oCRF | [^125^I]Tyr0-oCRF | FALSE |
| SECOND MESSENGERS | 12.98% | Nitric Oxide, NOS (Neuronal-Binding) | 2.63E-8 | NOARG (Nitro-L-Arginine) | [^3^H]NOARG | FALSE |
| NEUROTRANSMITTER RELATED | 10.87% | Nicotinic, Neuronal (a-BnTx insensitive) | 9.12E-11 | (+/-) Epibatidine | [^3^H]Epibatidine | FALSE |
| NEUROTRANSMITTER RELATED | 10.74% | GABA-B | 5.62E-7 | (+/-) Baclofen | [^3^H]CGP 54626A | FALSE |
| NEUROTRANSMITTER RELATED | 6.50% | Sigma, Non-selective | 3.57E-9 | Haloperidol | [^3^H]DTG | FALSE |
| NEUROTRANSMITTER RELATED | 6.25% | Glutamate, MK-801 Site (Ionotropic) | 1.24E-8 | (+)-MK-801 HMaleate | [^3^H]MK-801 | FALSE |
| ION CHANNELS | 6.07% | Potassium Channel, ATP-Sensitive | 7.81E-10 | Glibenclamide | [^3^H]Glibenclamide | FALSE |
| NEUROTRANSMITTER RELATED | 5.99% | Glutamate, NMDA Agonist Site (Ionotropic) | 6.81E-6 | NMDA | [^3^H]CGP 39653 | FALSE |
| BRAIN/GUT PEPTIDES | 4.50% | Endothelin, ET-B (h) | 2.88E-10 | Endothelin-1 | [^125^I]-Endothelin-1 | FALSE |
| BRAIN/GUT PEPTIDES | 2.96% | Angiotensin II, AT2 | 2.55E-10 | Angiotensin II (Human) | [^125^I]Tyr4-Angiotensin II | FALSE |
| ION CHANNELS | 2.07% | Potassium Channel, Ca2+ Act., VI | 2.05E-10 | Apamin | [^125^I]Apamin | FALSE |
| NEUROTRANSMITTER RELATED | 1.34% | Glutamate, AMPA Site (Ionotropic) | 9.07E-8 | (+/-) AMPA HBr | [^3^H]AMPA | FALSE |
| NEUROTRANSMITTER RELATED | 1.28% | Adenosine, Non-selective | 7.46E-9 | Neca | [^3^H]NECA | FALSE |
| NEUROTRANSMITTER RELATED | 0.09% | Serotonin, Non-selective | 6.94E-9 | Methysergide maleate | [^3^H] LSD | FALSE |
| BRAIN/GUT PEPTIDES | 0.09% | Cholecystokinin, CCK2 (CCKB) | 3.07E-10 | CCK-8 (sulfated) | [^125^I]CCK-8 | FALSE |
| NEUROTRANSMITTER RELATED | -0.22% | Glycine, Strychnine-sensitive | 2.81E-8 | Strychnine nitrate | [^3^H]Strychnine | FALSE |
| ENZYMES | -1.41% | Decarboxylase, Glutamic Acid | 1.17E-6 | AminoOxy acetic acid | [^14^C]Glutamic acid | FALSE |
| NEUROTRANSMITTER RELATED | -1.90% | Histamine, H1 | 7.33E-9 | Triprolidine | [^3^H]Pyrilamine | FALSE |
| NEUROTRANSMITTER RELATED | -13.63% | Glutamate, Kainate Site (Ionotropic) | 1.08E-8 | Kainic Acid | [^3^H]Kainic acid | FALSE |
| NEUROTRANSMITTER RELATED | -17.52% | Glutamate,NMDA,Glycine (Stry-insens Site) (Ionot | 3.15E-8 | MDL-105,519 | [^3^H]-MDL-105,519 | FALSE |

**Table** B**. Inhibition of hKir2.1 potassium channel activity with pentamidine isethionate.**

**Evaluated by the QPatch HT an automatic parallel patch clamp system.** The duration of exposure to each test concentration was 3 minutes.

| Concentration  (μM) | % hKir2.1 Inhibition  Mean (± SEM) % | Number of cells  n |
| --- | --- | --- |
| 0.001 | -2.9 (± 1.7) | 5 |
| 0.01 | 1.2 (± 1.4) | 5 |
| 0.1 | 1.4 (± 1.3) | 5 |
| 1 | 3.0 (± 2.7) | 5 |
| 10 | 12.3 (± 1.3) | 4 |

**Table C.** **A visual evaluation of the phase separation of Pluronics dispersions in pure water. Transparent is fully transparent. Opaque completely blocks light.** Slight indicates for slightly translucent (faintly white tint in the solution), and medium indicates obvious translucence.

| **P105**  **Wt%** | **F68**  **Wt%** | **L61**  **Wt%** | **Temperature**  **25°C** | **Temperature**  **30°C** | **Temperature**  **37°C** | **Temperature**  **40°C** | **Temperature**  **45°C** | **Temperature**  **50°C** |
| --- | --- | --- | --- | --- | --- | --- | --- | --- |
| 5 | 0 | 0.5 | Transparent | Transparent | Transparent | Transparent | Transparent | Transparent |
| 0 | 5 | 0.5 | Transparent | medium | opaque | opaque | opaque | opaque |
| 5 | 5 | 0.5 | Transparent | Transparent | Transparent | Transparent | Transparent | Transparent |
| 5 | 0 | 0.1 | Transparent | Transparent | Transparent | Transparent | Transparent | Transparent |
| 0 | 5 | 0.1 | Transparent | Transparent | slight | slight | slight | slight |
| 5 | 5 | 0.1 | Transparent | Transparent | Transparent | Transparent | Transparent | Transparent |
| 2.5 | 0 | 0.5 | Transparent | Transparent | Transparent | Transparent | Transparent | Transparent |
| 0 | 2.5 | 0.5 | Transparent | Medium | Opaque | Opaque | Opaque | Opaque |
| 2.5 | 2.5 | 0.5 | Transparent | Transparent | Transparent | Transparent | Transparent | Transparent |
| 2.5 | 0 | 0.1 | Transparent | Transparent | Transparent | Transparent | Transparent | Transparent |
| 0 | 2.5 | 0.1 | Transparent | Transparent | Slight | Medium | Medium | Medium |
| 2.5 | 2.5 | 0.1 | Transparent | Transparent | Transparent | Transparent | Transparent | Transparent |
| 1 | 0 | 0.1 | Transparent | Transparent | Transparent | Transparent | Transparent | Transparent |
| 0 | 1 | 0.1 | Transparent | Transparent | Medium | Opaque | Opaque | Opaque |
| 1 | 1 | 0.1 | Transparent | Transparent | Transparent | Transparent | Transparent | Transparent |
| 0.5 | 0 | 0.1 | Transparent | Transparent | Transparent | Transparent | Transparent | Transparent |
| 0 | 0.5 | 0.1 | Transparent | Transparent | Medium | Medium | Medium | Medium |
| 0.5 | 0.5 | 0.1 | Transparent | Transparent | Transparent | Transparent | Transparent | Transparent |
| 0.2 | 0 | 0.1 | Transparent | Transparent | Medium | Medium | Medium | Medium |
| 0 | 0.2 | 0.1 | Transparent | Transparent | Medium | Medium | Medium | Medium |
| 0.2 | 0.2 | 1 | Transparent | Transparent | Medium | Medium | Medium | Medium |
| 0 | 0 | 0.5 | Transparent | Opaque | Opaque | Opaque | Opaque | Opaque |
| 0 | 0 | 0.1 | Transparent | Slight | Opaque | Opaque | Opaque | Opaque |

**Table D.** **A visual evaluation of the phase separation of pluronics dispersions in saline. Transparent is fully transparent**. Opaque completely blocks light. Slight indicates for slightly translucent (faintly white tint in the solution), and medium indicates obvious translucence.

| **P105**  **Wt%** | **F68**  **Wt%** | **L61**  **Wt%** | **Temperature**  **25°C** | **Temperature**  **30°C** | **Temperature**  **37°C** | **Temperature**  **40°C** | **Temperature**  **45°C** | **Temperature**  **50°C** |
| --- | --- | --- | --- | --- | --- | --- | --- | --- |
| 5 | 0 | 0.5 | Transparent | Transparent | Transparent | Transparent | Transparent | Transparent |
| 0 | 5 | 0.5 | Transparent | Opaque | Opaque | Opaque | Opaque | Opaque |
| 5 | 5 | 0.5 | Transparent | Transparent | Transparent | Transparent | Transparent | Transparent |
| 5 | 0 | 0.1 | Transparent | Transparent | Transparent | Transparent | Transparent | Transparent |
| 0 | 5 | 0.1 | Transparent | Transparent | Slight | Slight | Slight | Slight |
| 5 | 5 | 0.1 | Transparent | Transparent | Transparent | Transparent | Transparent | Transparent |
| 2.5 | 0 | 0.5 | Transparent | Transparent | Transparent | Transparent | Transparent | Transparent |
| 0 | 2.5 | 0.5 | Transparent | Opaque | Opaque | Opaque | Opaque | Opaque |
| 2.5 | 2.5 | 0.5 | Transparent | Transparent | Transparent | Transparent | Transparent | Transparent |
| 2.5 | 0 | 0.1 | Transparent | Transparent | Transparent | Transparent | Transparent | Transparent |
| 0 | 2.5 | 0.1 | Transparent | Transparent | Medium | Medium | Medium | Medium |
| 2.5 | 2.5 | 0.1 | Transparent | Transparent | Transparent | Transparent | Transparent | Transparent |
| 1 | 0 | 0.1 | Transparent | Transparent | Transparent | Transparent | Transparent | Transparent |
| 0 | 1 | 0.1 | Transparent | Slight | Medium | Opaque | Opaque | Opaque |
| 1 | 1 | 0.1 | Transparent | Transparent | Transparent | Transparent | Transparent | Transparent |
| 0.5 | 0 | 0.1 | Transparent | Transparent | Transparent | Transparent | Transparent | Transparent |
| 0 | 0.5 | 0.1 | Transparent | Slight | Medium | Opaque | Opaque | Opaque |
| 0.5 | 0.5 | 0.1 | Transparent | Transparent | Transparent | Transparent | Transparent | Transparent |
| 0.2 | 0 | 0.1 | Transparent | Transparent | Medium | Medium | Medium | Medium |
| 0 | 0.2 | 0.1 | Transparent | Slight | Opaque | Opaque | Opaque | Opaque |
| 0.2 | 0.2 | 1 | Transparent | Transparent | Medium | Medium | Medium | Medium |
| 0 | 0 | 0.5 | Medium | Opaque | Opaque | Opaque | Opaque | Opaque |
| 0 | 0 | 0.1 | Transparent | Opaque | Opaque | Opaque | Opaque | Opaque |

**Table E**. **Stokes Radii of P105, P85 and F68 Micelles Obtained from DLS (1% w/w, 37°C).**

| **Pluronic_(aq)_** | **Radius**  **(nm)** | **Pluronic_(saline)_** | **Radius**  **(nm)** |
| --- | --- | --- | --- |
| F68 | 2.8 | F68 | 2.5 |
| P85 | 6.6 | P85 | 6.5 |
| P105 | 8.1 | P105 | 8.0 |
| [PTI]= 10^-6^ M |  | [PTI]= 10^-6^ M |  |
| F68 | 2.8 | F68 | 2.6 |
| P85 | 6.6 | P85 | 7.8 |
| P105 | 7.7 | P105 | 6.7 |
| PTI - 1:3 mass ratio |  | PTI - 1:3 mass ratio |  |
| F68 | 2.3 | F68 | 3.1 |
| P85 | 6.8 | P85 | 6.5 |
| P105 | 8.1 | P105 | 8.0 |

**Table F**. **The effect of P85, F68 and P105 on the apparent permeability of pentamidine isethionate across MDR1-MDCK cell monolayers in the basolateral to apical direction.** The apical to basolateral movement of pentamidine isethionate was below the limits of detection. The percentage recovery of pentamidine isethionate is also shown. Lucifer yellow permeation was below 0.5 x 10^-6^ cm/s in all experiments confirming the integrity of the monolayer. Transcellular marker (propranolol) and Pgp and BCRP substrate (prazosin) apparent permeability values are also shown.

| Drug | Concentration (%) | P_app_ A2B (10^-6^ cm/s) | | P_app_ B2A (10^-6^ cm/s) | | Mass balance A2B (%) | Mass balance B2A (%) |
| --- | --- | --- | --- | --- | --- | --- | --- |
|  |  | Replicate 1 | Replicate 2 | Replicate 1 | Replicate 2 |  |  |
| 10 μM propranolol^1^ | - | 34.1 | 28.0 | 41.4 | 41.6 | 81.5 | 84.4 |
| 10 μM prazosin^2^ | - | 2.48 | 1.96 | 69.5 | 70.7 | 92.0 | 92.0 |
| 10 μM pentamidine | - | - | - | 0.336 | 0.357 | 74.5 | 72.7 |
| 10 μM pentamidine | 0.01% P85 | - | - | 0.237 | 0.206 | 83.9 | 86.0 |
| 10 μM pentamidine | 0.1% P85 | - | - | 0.344 | 0.343 | 82.4 | 106.0 |
| 10 μM pentamidine | 0.5% P85 | - | - | 0.443 | 0.433 | 88.5 | 96.9 |
| 10 μM pentamidine | 0.01% P105 | - | - | 0.265 | 0.272 | 84.8 | 91.4 |
| 10 μM pentamidine | 0.1% P105 | - | - | 0.221 | 0.237 | 80.6 | 82.5 |
| 10 μM pentamidine | 0.5% P105 | - | - | 0.337 | 0.391 | 87.0 | 89.8 |
| 10 μM pentamidine | 0.01% F68 | - | - | 0.211 | 0.233 | 86.6 | 87.9 |
| 10 μM pentamidine | 0.1% F68 | - | - | 0.314 | 0.410 | 89.4 | 89.4 |
| 10 μM pentamidine | 0.5% F68 | - | - | 0.233 | 0.186 | 89.4 | 93.9 |

^1^ Transcellular maker that diffuses through cell membranes [11].
^2^ Pgp and BCRP substrate [12][13].

**Table G. The effect of Pluronic P85 on the accumulation of [^3^H(G)]pentamidine (15.7 nM) into brain tissues after 10 minutes of *in situ* perfusion.** All values have been corrected for vascular space by subtraction of the R_TISSUE_% for [^14^C(U)]sucrose from the R_TISSUE_% for [^3^H(G)]pentamidine. All values mean ± SEM.

| **Region** | **R_CORR TISSUE_%** | | | |
| --- | --- | --- | --- | --- |
|  | **[^3^H]pentamidine (n=8)** | **[^3^H]pentamidine**  **+ 0.01% P85 (n=8)** | **[^3^H]pentamidine**  **+ 0.1% P85 (n=5)** | **[^3^H]pentamidine**  **+ 0.5% P85  (n=8)** |
| **frontal cortex** | 4.29 (± 0.93) | 1.99 (± 0.18) | 2.31 (± 0.92) | 4.91 (± 0.98) |
| **caudate putamen** | 4.02 (± 1.03) | 1.58 (± 0.19) | 0.94 (± 0.92) | 2.81 (± 0.58) |
| **occipital cortex** | 4.03 (± 0.98) | 1.80 (± 0.34) | 1.93 (± 0.92) | 4.02 (± 1.03) |
| **hippocampus** | 4.37 (± 0.97) | 1.25 (± 0.33) | 0.94 (± 0.92) | 3.28 (± 1.06) |
| **corpus callosum** | 6.73 (± 2.65) | 4.77 (± 1.90) | 3.40 (± 1.51) | 3.71 (± 1.30) |
| **hypothalamus** | 5.56 (± 1.58) | 4.54 (± 0.87) | 1.82 (± 0.92) | 8.71 (± 2.49) |
| **thalamus** | 4.76 (± 0.91) | 1.42 (± 0.33) | 1.38 (± 0.92) | 5.13 (± 1.12) |
| **pons** | 7.16 (± 1.75) | 3.15 (± 0.64) | 2.36 (± 0.92) | 8.16 (± 2.11) |
| **cerebellum** | 3.82 (± 0.64) | 1.59 (± 0.14) | 1.66 (± 0.92) | 4.69 (± 1.02) |
| **homogenate** | 4.61 (± 0.94) | 2.19 (± 0.40) | 1.41 (± 0.20) | 4.59 (± 1.06) |
| **supernatant** | 1.99 (± 0.45) | 0.58 (± 0.10) | 0.63 (± 0.09) | 1.64 (± 0.30) |
| **pellet** | 12.54 (± 1.74) | 8.43 (± 0.92) | 9.63 (± 3.22) | 14.13 (± 2.71) |
| **pineal gland** | 284.7 (± 81.4) | 285.8 (± 56.4) | 166.2 (± 19.9) | 372.6 (± 41.0) |
| **choroid plexus** | 490.9 (± 103.1) | 432.7 (± 84.3) | 836.3 (± 150.6) | 703.80 (± 150.6) |
| **pituitary gland** | 182.75(± 29.6) | 450.74(± 88.6) | 569.21(± 51.3) | 517.48(± 114.0) |

**Table H.** **The effect of Pluronic P105 on the accumulation of [^3^H(G)]pentamidine (15.7 nM) into brain parenchyma after 10 minutes of *in situ* perfusion.** All values have been corrected for vascular space by subtraction of the R_TISSUE_% for [^14^C(U)] sucrose from the R_TISSUE_% for [^3^H(G)]pentamidine.

| **Region** | **R_CORR TISSUE_%** | | | |
| --- | --- | --- | --- | --- |
|  | **[^3^H]pentamidine (n=8)** | **[^3^H]pentamidine+**  **0.01% P105 (n=4)** | **[^3^H]pentamidine+**  **0.1% P105 (n=8)** | **[^3^H]pentamidine+**  **0.5% P105 (n=4)** |
| **frontal cortex** | 4.29 (± 0.93) | 3.70 (± 0.36) | 3.36 (± 0.78) | 1.96 (± 1.44) |
| **caudate putamen** | 4.02 (± 1.03) | 3.47 (± 0.61) | 2.36 (± 0.48) | 2.46 (± 0.87) |
| **occipital cortex** | 4.03 (± 0.98) | 3.95 (± 0.99) | 2.83 (± 0.49) | 1.90 (± 0.58) |
| **hippocampus** | 4.37 (± 0.97) | 3.22 (±0.92) | 3.20 (± 0.69) | 0.68 (± 0.36) |
| **corpus callosum** | 6.73 (± 2.65) | 10.79 (± 4.21) | 6.46 (± 1.10) | 3.62 (± 1.37) |
| **hypothalamus** | 5.56 (± 1.58) | 6.36 (± 2.16) | 3.95 (± 0.59) | 2.77 (± 1.04) |
| **thalamus** | 4.76 (± 0.91) | 4.98 (± 1.56) | 2.44 (± 0.35) | 4.82 (± 1.05) |
| **pons** | 7.16 (± 1.75) | 5.52 (± 2.87) | 5.83 (± 1.54) | 1.51 (± 1.43) |
| **cerebellum** | 3.82 (± 0.64) | 2.42 (± 0.44) | 3.23 (± 0.56) | 4.03 (± 0.56) |
| **homogenate** | 4.61 (± 0.94) | 2.75 (± 0.59) | 3.22 (± 0.60) | 3.33 (± 0.36) |
| **supernatant** | 1.99 (± 0.45) | 1.12 (± 0.28) | 1.25 (± 0.25) | 1.31 (± 0.20) |
| **pellet** | 12.54 (± 1.74) | 9.49 (± 0.72) | 16.73 (± 5.17) | 6.59 (± 0.74) |
| **pineal gland** | 284.7 (± 81.4) | 326.2 (± 10.5) | 266.7 (± 28.3) | 315.9 (± 17.4) |
| **choroid plexus** | 490.9 (± 103.1) | 289.1 (± 74.3) | 474.9 (± 74.6) | 402.6 (± 166.1) |
| **pituitary gland** | 182.75(± 37.5) | 425.16(± 63.9) | 437.78(± 105.4) | 458.99(± 166.1) |

**Table I.** **Accumulation of ^3^H-pentamidine (15.7 nM) after 10 minutes perfusion with or without pluronic F68 (not corrected for vascular space; Control A and 0.01% and 0.1% F68 experiments were carried out using MP Biomedicals dextran.** Control B and 0.5% F68 experiments were carried out using VWR dextran).

| ^3^H-pentamidine | **R_TISSUE/PLASMA_%** | | | **(mean±SEM)** |  |
| --- | --- | --- | --- | --- | --- |
| **Region** | **Control A [^3^H]pentamidine (n=5)** | **[^3^H]pentamidine**  **+ 0.01% F68 (n=5)** | **[^3^H]pentamidine**  **+ 0.1% F68 (n=5)** | **Control B [^3^H]pentamidine**  **(n=5)** | **[^3^H]pentamidine**  **+ 0.5% F68 (n=5)** |
| **Frontal cortex** | 4.88 (± 1.18) | 4.48 (± 0.37) | 3.57 (± 0.63) | 6.23 (± 0.92) | 5.73 (± 0.73) |
| **Caudate putamen** | 4.08 (± 0.84) | 2.94 (± 0.28) | 2.38 (± 0.28) | 5.40 (± 1.00) | 3.19 (± 0.42) |
| **Occipital cortex** | 4.56 (±0.87) | 3.89 (± 0.54) | 3.95 (± 0.42) | 5.67 (± 0.99) | 4.18 (± 0.77) |
| **Hippocampus** | 3.48 (± 0.94) | 2.91 (± 0.26) | 2.51 (± 0.10) | 5.60 (± 1.43) | 3.50 (± 0.48) |
| **Corpus callosum** | 5.32 (± 1.47) | 4.45 (±0.48) | 3.26 (± 0.49) | 9.17 (± 2.67) | 6.21 (± 1.42) |
| **Hypothalamus** | 5.96 (± 1.23) | 4.95 (± 1.17) | 3.90 (± 0.60) | 8.14 (± 1.75) | 6.50 (± 1.14) |
| **Thalamus** | 4.34 (± 0.83) | 3.16 (± 0.38) | 3.24 (± 0.35) | 5.95 (± 0.92) | 3.96 (± 0.36) |
| **Pons** | 7.81 (± 0.90) | 8.41 (± 1.26) | 3.60 (± 0.64) | 10.72 (± 1.82) | 8.25 (± 2.14) |
| **Cerebellum** | 5.28 (± 0.56) | 4.77 (± 0.59) | 4.59 (± 0.63) | 6.54 (± 0.70) | 5.95 (± 0.74) |
| **Homogenate** | 4.03 (± 0.78) | 4.21 (± 0.34) | 4.42 (± 0.76) | 6.20 (± 0.97) | 6.03 (± 0.57) |
| **Supernatant** | 2.54 (± 0.39) | 2.39 (± 0.29) | 2.13 (± 0.40) | 3.00 (± 0.43) | 2.97 (± 0.28) |
| **Pellet** | 4.11 (± 1.24) | 7.50 (± 0.99) | 8.28 (± 2.47) | 13.19 (± 1.79) | 15.14 (± 3.19) |
| **Pineal gland** | 367.59 (± 39.97) | 210.80 (± 33.30) | 210.41 (± 48.84) | 361.76 (± 79.03) | 350.80 (± 58.07) |
| **Choroid plexus** | 385.74 (± 24.35) | 357.71 (± 58.50) | 376.66 (± 74.84) | 535.95 (± 107.86) | 526.78 (± 197.05) |
| **Pituitary gland** | 251.21 (± 66.80) | 167.83 (± 21.49) | 223.56 (± 19.43) | 203.27(± 32.85) | 497.44 (± 133.74) |

**Table J. Accumulation of [^14^C]sucrose after 10 minutes perfusion with or without Pluronic F68; Control A and 0.01% and 0.1% F68 experiments were carried out using MP Biomedicals dextran.** Control B and 0.5% F68 experiments were carried out using VWR dextran).

| ^14^C-sucrose | **R_TISSUE/PLASMA_%** | | | **(mean±SEM)** |  |
| --- | --- | --- | --- | --- | --- |
| **Region** | **Control A [^14^C]sucrose (n=5)** | **[^14^C]sucrose**  **+0.01% F68 (n=5)** | **[^14^C]sucrose**  **+ 0.1% F68 (n=5)** | Control B **[^14^C]sucrose** (n=5) | **[^14^C]sucrose**  **+ 0.5% F68** (n=5) |
| **Frontal cortex** | 2.25 (± 0.45) | 2.10 (± 0.24) | 1.72 (± 0.33) | 1.94 (± 0.23) | 2.28 (± 0.43) |
| **Caudate putamen** | 2.01 (± 0.37) | 1.21 (± 0.21) | 1.05 (± 0.18) | 1.38 (± 0.12) | 1.17 (± 0.33) |
| **Occipital cortex** | 1.85 (± 0.38) | 1.64 (± 0.34) | 1.64 (± 0.49) | 1.64 (± 0.11) | 1.57 (± 0.41) |
| **Hippocampus** | 1.52 (± 0.37) | 1.22 (± 0.21) | 0.99 (± 0.12) | 1.23 (± 0.09) | 1.25 (± 0.29) |
| **Corpus callosum** | 2.88 (± 0.65) | 1.88 (± 0.34) | 1.38 (± 0.49) | 1.30 (± 0.32) | 1.80 (± 0.42) |
| **Hypothalamus** | 3.72 (± 0.88) | 2.04 (± 0.39) | 2.45 (± 0.53) | 2.58 (± 0.34) | 2.23 (± 0.22) |
| **Thalamus** | 1.66 (± 0.27) | 1.06 (± 0.22) | 1.12 (± 0.17) | 1.19 (± 0.14) | 1.18 (± 0.16) |
| **Pons** | 4.02 (± 0.49) | 4.23 (± 0.68) | 3.04 (± 0.91) | 3.56 (± 0.34) | 3.41 (± 0.53) |
| **Cerebellum** | 2.82 (± 0.41) | 2.50 (± 0.45) | 2.81 (± 0.83) | 2.72 (± 0.32) | 2.53 (± 0.38) |
| **Homogenate** | 1.80 (±0.34) | 1.63 (±0.31) | 1.76 (±0.54) | 1.59 (±0.15) | 1.77 (± 0.19) |
| **Supernatant** | 1.35 (±0.17) | 1.18 (±0.20) | 1.38 (±0.50) | 1.01 (±0.09) | 1.39 (± 0.26) |
| **Pellet** | 0.41 (±0.14) | 0.48 (±0.07) | 0.55 (±0.15) | 0.65 (±0.07) | 0.79 (± 0.09) |
| **Pineal gland** | 78.85 (±11.23) | 43.97 (±11.10) | 34.64 (±7.88) | 77.04 (±16.38) | 62.65 (± 6.00) |
| **Choroid plexus** | 54.64 (±15.07) | 42.36 (±10.69) | 37.01 (±8.46) | 45.06 (±7.07) | 47.70 (± 12.87) |
| **Pituitary gland** | 48.66 (±10.98) | 16.85 (±3.19) | 17.94 (±1.57) | 20.52 (±3.86) | 35.09 (± 4.82) |

**Table K. Accumulation of [^3^H]pentamidine after 30 minutes perfusion with or without pluronic F68**. (Not corrected for vascular space).

| **[^3^H]pentamidine** | **R_TISSUE/PLASMA_% (mean ± SEM)** | | | |
| --- | --- | --- | --- | --- |
| **Region** | **Control  (15.7 nM pent) (n=5)** | **0.01% F68 +  (15.7 nM pent) (n=5)** | **0.1% F68 + (15.7 nM pent) (n=4)** | **0.5% F68 + (15.7 nM pent) (n=5)** |
| **Frontal cortex** | 10.35 (± 0.84) | 9.00 (± 1.47) | 11.91 (± 1.35) | 17.54 (± 3.58) |
| **Caudate putamen** | 8.65 (± 0.28) | 6.90 (± 1.58) | 10.41 (± 1.18) | 13.07 (± 3.45) |
| **Occipital cortex** | 9.52 (± 1.08) | 8.80 (± 1.47) | 12.14 (± 1.62) | 15.85 (± 2.71) |
| **Hippocampus** | 8.19 (± 0.30) | 6.05 (± 1.20) | 7.93 (± 1.20) | 10.65 (± 2.55) |
| **Corpus callosum** | 8.19 (± 1.97) | 2.02 (± 1.22) | 9.47 (± 0.94) | 15.72 (± 6.78) |
| **Hypothalamus** | 18.49 (± 4.17) | 8.90 (± 1.42) | 11.17 (± 1.42) | 18.73 (± 4.67) |
| **Thalamus** | 9.94 (± 1.95) | 7.99 (± 1.06) | 11.08 (± 1.06) | 13.52 (± 3.10) |
| **Pons** | 15.16 (± 1.48) | 17.61 (± 3.70) | 20.36 (± 3.70) | 20.43 (± 5.67) |
| **Cerebellum** | 12.92 (± 1.10) | 9.38 (± 2.47) | 14.44 (± 2.47) | 17.28 (± 3.54) |
| **Homogenate** | 10.18 (± 0.92) | 9.63 (± 0.93) | 11.19 (± 1.62) | 14.45 (± 3.93) |
| **Supernatant** | 5.54 (± 0.37) | 3.77 (± 0.94) | 6.63 (± 0.52) | 6.45 (± 1.35) |
| **Pellet** | 16.45 (± 2.76) | 18.16 (± 2.79) | 22.15 (± 4.44) | 18.42 (± 2.97) |
| **Pineal gland** | 664.25 (± 82.42) | 341.30 (± 39.57) | 342.95 (± 92.02) | 525.94 (± 122.85) |
| **Choroid plexus** | 1241.60 (± 92.86) | 835.26 (± 260.20) | 944.85 (± 235.90) | 1844.80 (± 493.51) |
| **Pituitary gland** | 394.51 (± 52.42) | 324.42 (± 44.06) | 434.10 (± 59.56) | 881.90 (± 170.25) |
|  |  |  |  |  |

**Table L. Accumulation of [^14^C]sucrose (B) after 30 minutes perfusion with or without pluronic F68.** (Not corrected for vascular space).

| **^14^C-sucrose** | **R_TISSUE/PLASMA_% (mean ± SEM)** | | | |
| --- | --- | --- | --- | --- |
| **Region** | **Control  (15.7 nM pent) (n=5)** | **0.01% F68 +  (15.7 nM pent) (n=5)** | **0.1% F68 + (15.7 nM pent) (n=4)** | **0.5% F68 + (15.7 nM pent) (n=5)** |
| **Frontal cortex** | 2.24 (± 0.29) | 5.02 (± 2.36) | 3.26 (± 0.38) | 5.42 (± 1.59) |
| **Caudate putamen** | 1.48 (± 0.18) | 4.12 (± 2.18) | 3.57 (± 0.44) | 4.02 (± 0.90) |
| **Occipital cortex** | 1.72 (± 0.12) | 3.70 (± 1.59) | 3.15 (± 0.33) | 4.26 (± 1.12) |
| **Hippocampus** | 1.32 (± 0.17) | 2.29 (± 1.22) | 2.79 (± 0.36) | 3.29 (± 1.12) |
| **Corpus callosum** | 1.67 (± 0.35) | 1.12 (± 2.52) | 4.21 (± 0.48) | 4.39 (± 2.08) |
| **Hypothalamus** | 3.50 (± 0.35) | 1.12 (± 0.99) | 4.21 (± 0.48) | 4.39 (± 2.08) |
| **Thalamus** | 1.68 (± 0.68) | 1.81 (± 1.58) | 4.21 (± 0.21) | 5.88 (± 1.04) |
| **Pons** | 4.29 (± 0.22) | 3.20 (± 2.06) | 3.09 (± 1.18) | 3.67 (± 1.49) |
| **Cerebellum** | 3.10 (± 0.26) | 5.57 (± 1.42) | 6.13 (± 0.99) | 7.21 (± 1.96) |
| **Homogenate** | 2.14 (± 0.23) | 2.60 (± 0.87) | 3.02 (± 0.52) | 3.05 (± 1.09) |
| **Supernatant** | 1.21 (± 0.17) | 2.11 (± 0.84) | 2.31 (± 0.26) | 2.53 (± 0.64) |
| **Pellet** | 1.09 (± 0.33) | 1.19 (± 0.30) | 1.43 (± 0.06) | 1.13 (± 0.18) |
| **Pineal gland** | 65.85 (± 8.08) | 30.21 (± 8.14) | 52.42 (± 4.23) | 74.63 (± 24.42) |
| **Choroid plexus** | 99.54 (± 15.32) | 66.37 (± 7.52) | 76.84 (± 13.07) | 116.86 (± 31.26) |
| **Pituitary gland** | 39.02 (± 5.27) | 18.41 (± 5.34) | 30.35 (± 5.23) | 65.21 (± 13.10) |

**References**

1. Safety Assessment of Poloxamers 101, 105, 108, 122, 123, 124, 181, 182, 183, 184, 185, 188, 212, 215, 217, 231, 234, 235, 237, 238, 282, 284, 288, 331, 333, 334, 335, 338, 401, 402, 403, and 407, Poloxamer 105 Benzoate, and Poloxamer 182 Dibenzoate as Use. Int J Toxicol. 2008;27: 93–128. doi:10.1080/10915810802244595

2. Kozlov MY, Melik-Nubarov NS, Batrakova E V., Kabanov A V. Relationship between Pluronic Block Copolymer Structure, Critical Micellization Concentration and Partitioning Coefficients of Low Molecular Mass Solutes. Macromolecules. 2000;33: 3305–3313. doi:10.1021/ma991634x

3. Heenan, R K; King, S.M., Osborn, R., Stanley HB. COLETTE Users Guide, Rutherford Appleton Laboratory Report. Rutherford Applet Lab Rep. 1989; 89–128.

4. King, S.M., Heenan RK. Using COLETTE. Rutherford Applet Lab Rep. 1995;95.

5. SasView. Schmitt, U. et al. (2012) ‘In vitro P-glycoprotein efflux inhibition by atypical antipsychotics is in vivo nicely reflected by pharmacodynamic but less by pharmacokinetic changes’, Pharmacology Biochemistry and Behavior, 102(2), pp. 312–320. doi: 10.1016/; [cited 23 Nov 2014]. Available: http://www.sasview.org/

6. Neun BW, Dobrovolskaia MA. Method for analysis of nanoparticle hemolytic properties in vitro. Methods Mol Biol. 2011;697: 215–24. doi:10.1007/978-1-60327-198-1_23

7. Triguero D, Buciak J, Pardridge WM. Capillary depletion method for quantification of blood-brain barrier transport of circulating peptides and plasma proteins. J Neurochem. 1990;54: 1882–8.

8. Sanderson L, Khan A, Thomas S. Distribution of suramin, an antitrypanosomal drug, across the blood-brain and blood-cerebrospinal fluid interfaces in wild-type and P-glycoprotein transporter-deficient mice. Antimicrob Agents Chemother. 2007;51: 3136–3146.

9. Shaik N, Giri N, Elmquist WF. Investigation of the micellar effect of pluronic P85 on P-glycoprotein inhibition: cell accumulation and equilibrium dialysis studies. J Pharm Sci. 2009;98: 4170–90. doi:10.1002/jps.21723

10. Batrakova E V., Han H-Y, Alakhov VY, Miller DW, Kabanov A V. Effects of Pluronic Block Copolymers on Drug Absorption in Caco-2 Cell Monolayers. Pharm Res. 1998;15: 850–855. doi:10.1023/A:1011964213024

11. Artursson P. Epithelial Transport Of Drugs In Cell Culture. I: A Model For Studying The Passive Diffusion Of Drugs Over Intestinal Absorbtive (Caco-2) Cells. J Pharm Sci. 1990;79: 476–482. doi:10.1002/jps.2600790604

12. Cisternino S. Expression, Up-Regulation, and Transport Activity of the Multidrug-Resistance Protein Abcg2 at the Mouse Blood-Brain Barrier. Cancer Res. 2004;64: 3296–3301. doi:10.1158/0008-5472.CAN-03-2033

13. Enokizono J, Kusuhara H, Ose A, Schinkel AH, Sugiyama Y. Quantitative Investigation of the Role of Breast Cancer Resistance Protein (Bcrp/Abcg2) in Limiting Brain and Testis Penetration of Xenobiotic Compounds. Drug Metab Dispos. 2008;36: 995–1002. doi:10.1124/dmd.107.019257
