# Supplementary material for: Drug reformulation for a neglected disease. The NANOHAT project to develop a safer more effective sleeping sickness drug
Source: PLoS Negl Trop Dis. 2021 Apr 15;15(4):e0009276. doi: 10.1371/journal.pntd.0009276 (PMC8078842; doi:10.1371/journal.pntd.0009276)
Supplement: Attachment — Submitted filename: Reviewer2_PNTD-D-20-00526.docx [file pntd.0009276.s002.docx]

**For the Editor:**

**PNTD-D-20-00526.**

**Title:** Drug reformulation for a neglected disease.  The NANOHAT project to develop a safer more effective sleeping sickness drug.

**Authors:** Lisa Sanderson et al.

**Background:** The use of toxic and antiquated drugs coupled with the increased incidence of drug resistance clearly underscores the need for new chemotherapeutic agents and/or approaches against human African trypanosomiasis (HAT). While it is commonly believed that small molecule drugs readily cross the BBB, the presence of tight junctions (TJs) effectively prevents 98% of small molecules and 100% of large drugs >400-500 Da from crossing the BBB. Some molecules can cross by lipid-mediated free diffusion if they are both lipid soluble and are <400 Da in size. Others cross by catalyzed transport via endogenous BBB transporters. The BBB can also metabolize drugs (i.e. glutamyl transamidase can detoxify glutathione-bound molecule) or pump them back into the blood by the apically located multi-drug resistance (MDR) efflux pumps: i.e. adenosine triphosphate (ATP)-binding cassette (ABC) transporters. There there are 48 members in this protein family in humans involved in transporting lipids, sterols, peptides, toxins, and ions. Of these, P-glycoprotein (P-gp or ABCB1) is the most characterized MDR transporter. Remarkably, there is presently not a single FDA HAT drug that penetrates the brain poorly that has been further chemically altered to improve brain penetration enabling maintained pharmacological activity and drug approval.

**What the authors want to do:** The authors have focused on a drug first reported in 1939, pentamidine (4,4’-diamidino,1,5-diphenoxy pentane), a diamidine usually used to treat stage 1 HAT (*T. b. gambiense*) when the parasites are largely found in the blood. For the most part, the drug is generally considered ineffective once the parasite has invaded the CNS because pentamidine is unable to cross the blood-brain (BBB) due to its removal by efflux transporters. One of the lesser toxic anti-HAT drugs, often dose-limiting factors due to peripheral side effects (hypoglycemia and diabetes mellitus) that can lead to incomplete treatment. Interestingly, there was some evidence a few years ago for clinical benefit from pentamidine use for some HAT patients with ‘early late stage’ infections, where the parasite has crossed the blood-brain barrier (BBB) but not yet fully penetrated the brain parenchyma [1]. To their credit, the authors have taken an ambitious nanotechnological approach to create and evaluate the physicochemical and structural characteristics of formulations of pentamidine with Pluronic micelles [amphiphilic triblock copolymers of poly(ethylene oxide) (PEO) and poly(propylene oxide) (PPO) with PEO-PPO-PEO structure]. that spontaneously self-assemble to encapsulate the drug. In addition to prolonging the drug’s circulation time in blood, it was hypothesized that the new pentamidine/Pluronic formulations would enhance passage of the drug across the BBB. How this might work is provided in their **Supplement Fig 1S**. In this example, Pluronic®P85 inhibits-mediated efflux (e.g. P-gp and MRP transport) by two mechanisms; the first through membrane fluidization and the second through transient ATP depletion. These effects are believed to be mediated by unimers (single polymer chains) [2-4]. The hypothesis/objective is that a polymeric micelle formulation of pentamidine with Pluronic block copolymers in isotonic buffered saline that will block drug efflux mechanisms thus allowing enhanced delivery of pentamidine across the Blood-Brain Barrier (BBB) into the brain. Using an ambitious, complex, supposedly iterative screening process (**Fig 1, this manuscript)** relying on methods derived from various biophysical, pharmaceutical and biological disciplines the goal was to find the optimal formulation end product ready to be taken for the final stage of the study pre-clinical testing for the treatment of Stage 2 HAT. The authors concluded that they had “…successfully identified Pluronic-pentamidine formulations that harbour trypanocidal activity and do not increase the safety concerns centrally or peripherally (over unformulated pentamidine)…” but were unable “…to significantly enhance brain exposure of pentamidine using the Pluronic (F68, P85 or P105) within a reasonable time frame and existing budget. We therefore drew the study to a close at milestone 2 (Fig 1).” It is a shame that the study did not go to completion as planned**.** But this Reviewer believes that even with more time to complete all milestone that the study as written would end the same way.

**Supplement Fig S1. [A]** Pentamidine taken up by the capillary endothelial cell is **[B]** efficiently returned back into the blood via P-gp and MRP mediated mechanisms.

**But these goals were not achieved. Why?. To understand why requires an understanding of BBB function.** In order to supply about 100 billion neurons the power they need the human brain consumes about 15-20 watts of power [5]. The brain is also critically dependent on the vasculature of the BBB to provide 15-20% of the body’s cardiac oxygen and glucose and other nutrients necessary to maintain brain function. *In the broadest context, the BBB can be considered to be the microvascular endothelial cells (MEC) that line the arterioles, capillaries and venules in the brain.* Brain MEC express stringent tight junctions (TJs) with differential complexities reflecting function. While similar in architecture across species, the brain microvasculature differs from other capillary networks in that it tightly regulates transport into the brain and exhibit significant plasticity in response to abnormal physiological conditions. Lacking pinocytic activity, brain MEC do enact bona fide transcytosis: i.e. blood glucose and insulin brain delivery through Glucose transporter 1 and the insulin receptor, respectively. Furthermore, fluid phase cellular uptake and paracellular transport insulin in brain (especially in non-brain MEC) at supraphysiological insulin concentrations is possible. Significant biochemical differences in expression levels of transporters and pumps unique to the human BBB have been documented. An integral component of the neurovascular unit (NVU), the BBB is responsible maintaining homeostatic signaling among the neuronal, glial and vascular endothelial cellular compartments, all important for maintaining normal brain function. Conversely, dysfunction in any component of the NVU affects overall function, thus contributing to disease. *The simple view of the BBB as a mere mechanical barrier is misleading without any realization of its limitations or the nature of the functional mechanisms that may be aﬀected*. A most important point, *the critical role of the BBB in vascular health and disease is exquisitely linked to changes in blood composition and shear-stress (SS) flow dynamics.* In the brain, blood flow starts off as pulsatile in arterioles and decreases in intensity as it flows from capillaries into venules where it can be either pulsatile or steady laminar i.e. strength of flow and flow patterns (pulsatile uniform, pulsatile disturbed, steady laminar) that brain MECs are exposed to. Dramatic changes in vasculature responses to SS can occur. All are critically linked as triggers of cellular/molecular responses that constantly change with time; i.e. within seconds (GPCR/ion channel activation), hours (cytoskeletal rearrangements, NF-κβ), and hours to days (cell proliferation, cell alignment).

**Major concerns:**

**The blood-brain studies:** These authors are well-versed in the appropriate use in vitro BBB models. They had previously reported a role for Organic Cation Transporter 1 (OCT1) involvement in pentamidine transport at the human and mouse BBB using hCMEC/D3 and bEnd.3 cell line monolayers [6]. So it is a total mystery why **in lines 470-476,** the authors say that *“…Several in vitro BBB models were evaluated…, before selecting the MDR1-MDCK cells…Madin-Darby canine kidney (MDCK) cells with the MDR1 gene, the gene encoding for the human efflux protein, P-glycoprotein (P-Gp)”,* because *“…Using MDR1-MDCK cells avoids the complexities of multiple transporters by focusing specifically on P-gp.”* From the start the authors tried to cherry pick their model to suit their hypothesis (Fig. 1S above). In the end, this challenges the physiologic worthiness of the data obtained from all the experiments using this model system. This epithelial cell overexpressing P-gp model may be cheap and easy to standardize, and it expresses non-physiologically high levels of the transporter but does not take into account the profound biological/functional differences between epithelial and endothelial cells especially MEC from human brain [7]. Although lacking shear-stress and lacking the support of other components of the NVU, models using brain endothelial cells that are readily available are still inexpensive. For P-gp target validation or drug permeability BBB monolayer with or without co-culture would still have been preferred model for study. The higher ATP levels in brain ECs would have played a role in how good Pluronics as P-gp inhibitors were.

The inappropriateness of the MDR1-MDCK model is alluded to from their *in situ* perfusion studies as they are (see next section). From lines 1129-1132, we know from their previous in vivo pentamidine efflux studies that P-gp was demonstrate studies [8], but they could not demonstrate any increase in formulated pentamidine movement across the MDR1-MDCK model in any direction compared with unformulated drug. Does this negate a role for P-gp and thus question the validity of their hypothesis overall, as others have found that P-gp plays no role in the efflux process [9].This is in line with their conclusions drawn for their flawed (see below) in situ brain perfusion studies in mice over 10 mins (lines 1136-1140) in which they *“…showed that the P85, P105 and F68 formulations have a tendency to actually prevent uptake of pentamidine into brain tissue and/or vascular endothelial cells, which constitute an intact BBB. This may be related to interactions of the Pluronics with influx transporters for pentamidine (e.g. OCT1), although our in vitro BBB studies did not indicate that the pentamidine permeability was affected by the presence of F68, P85 and P105 (0.01% and 0.1%) in either direction*. The reason their MDR1-MDCK model failed to show this is because IT IS NOT A BBB MODEL IN THE FIRST PLACE.

**Brain perfusion studies**: There are questions in the value of the data obtained in doing brain perfusion/capillary depletion studies under less than ideal physiologic flow conditions especially when the in vivo intravenous pharmacokinetic studies, a reference for brain uptake studies under fully physiological conditions showed no statistical differences in brain localization/entry kinetics for any p68 drug formulation and free pentamidine. For sure, complementing the intravenous analysis using brain perfusion platforms offers some unique advantages but these are complex and time consuming for what more information could have been extracted. In the end the brain perfusion studies also failed to find differences in brain localization. While the drug perfusion studies appear to be done properly in the classical sense, for future experimentation perfusion condition(s) should take into account physiologic SS flow and flow patterns based on normal (mouse?) heartbeat and flow rates. The data set obtained will certainly change, but whether the final outcome will be altered is something to think about.

**Overall,** considering the toxic nature of even the safest of anti-African trypanosome drugs and the relative safety of Pluronic base drugs in general, it would have made more sense to skip the experimentally complex F68 pluronic/pentamidine brain localization perfusion studies and directly test the F68 formulations in trypanosome infection-based murine models of CNS HAT; i.e. Kaplan-Meier study or drug studies based on live IVM imaging using luciferase-labeled parasites [10].

**Some notes (no particular order)**

**Notes on Pluronic formulations for pentamidine delivery**: By customizing the lengths of the polymer blocks, pluronics with slightly different properties can be created. The naming of pluronic copolymers is based their physical form at room temperature, defined by the first letter: L = liquid, P = paste, F = flake (solid). The authors initially choose to focus on 4 pluronic blocks—L61, F68, P85 and P105 based on well-established phase behavior and cloud points, their solubility in water and saline solutions at both 24°C and 37°C and, most important to their hypothesis, had P-gp inhibitor properties. With a very low cloud point at 24° and the inability to improve to solubility of highly hydrophobic L61 even in mixture with highly hydrated F68, was reason of L61’s elimination from the list (**Table S3 and S4**).

Surprisingly, Pluronics P85 and P105 alone (no pentamidine) at exceedingly low concentrations (**Table 8**) were highly trypanotoxic. Regardless whether or not these two Pluronic can cross the BBB, they may find usefulness as a next-generation drugs for Stage 1 gambiense HAT. Nonetheless, unlike P68 formulations, P85 and P105 pentamidine formulations were eliminated presumably because of **Figure 3** toxicity issues regarding insulin secretion. However, a closer look at the data may have overlooked a potentially protective arm for F68 formulations (see below).

**The trypanosome toxicity studies** shown brought up RED FLAGS. 10 nM pentamidine is trypanocidal. Yet **Table 9** that unformulated pentamidine at 015 nm is trypanocidal activity (kills 92.7%) of 0.15nM (100x lower than literature). Something wrong here??? Everyone else is wrong??? The lowest conc of pentamidine in **Table 9** is 5.7 x 10^-6^ µM, which is 5.7 pM. There should be a 0 uM drug column to have better idea of % tryps killed in present of Pluronic alone: Maybe their Tbb 427 strain was an ultrasensitive one? In reality, for future reference the data would have been more meaningful had the tests been done using *T b gambiense* parasites.

**MIN6 -cell insulin secretion studies:** More to the point, the effect pentamidine and Pluronics on MIN6 -cell insulin secretion shown in Figure 3 were based only on 3 very high pentamidine concentrations 100 times, 1000 times and 10000 times the trypanocidal dose. From ***lines 832-836,*** it is clear that while all concentrations of P85 and P105 strongly suppressed of insulin secretion from MIN6 β-cells. However, compared to free pentamidine the F68 formulations only induced insulin secretion suppression at concentrations ≥0.1% w/v. Not much is said about the 20% increase in insulin secretion at the 1 μM free/F68 pentamidine dose!!! This reviewer suggests that this study should be redone using sub to high nM of low μM range as there may be some hidden message (protective mechanism?) here.

**CNS side effects: *Lines 193-197*** states ‘…A CNS side effect panel was custom designed and binding assays performed by Perkin-Elmer Science Discovery Systems…Testing was performed at a single concentration of 1 μM (100-times the trypanocidal concentration), with follow up concentration-response curves in any assay where there was greater than 70% inhibition to determine an inhibition constant (Ki)***.*** And yet ***Lines 654-657*** states that ‘...A wide ligand profiling screen was carried out against 40 CNS targets (Perkin Elmer customised CNS screening; listed in **Table S1**), testing at a single concentration of 10 μM (1000-times the trypanocidal concentration), with follow up concentration-response curves in any assay where there was greater than 70% inhibition.’ First of all, there is no real explanation on how Perkin-Elmer did the actual assays and the context for the basis of the 40 CNS targets. Clear this was a justification for a quick CNS toxicity assay rather than doing the tests properly. The correct way would have been to serially dilute the formulations starting from sub-nM range based on pentamidine content upwards, certainly no higher than 1 μM.

**References**

1.doi: 10.4269/ajtmh.1996.55.586.

2.	doi: 10.1002/jps.21723.

3.	doi: 10.1023/a:1011964213024.

4.	doi: 10.1023/a:1011942814300.

5.	doi: 10.3389/fneng.2013.00007.

6.	doi: 10.1371/journal.pone.0173474.

7.	doi: 10.2147/dddt.S218708.

8.	doi: jpet.108.149872 [pii]10.1124/jpet.108.149872.

9.	doi: 10.1128/AAC.02605-14.

10.	doi: 10.1371/journal.pntd.0002384.
