# Supplementary material for: Drug reformulation for a neglected disease. The NANOHAT project to develop a safer more effective sleeping sickness drug
Source: PLoS Negl Trop Dis. 2021 Apr 15;15(4):e0009276. doi: 10.1371/journal.pntd.0009276 (PMC8078842; doi:10.1371/journal.pntd.0009276)
Supplement: Attachment — Submitted filename: RESPONSE TO REVIEWERS COMMENTS.docx [file pntd.0009276.s003.docx]

**EDITOR**

**We recognise that you have as a team performed a thorough analyses of this approach to drug treatment, which has failed. We consider this important for the community to know. PLoS NTD readership will be lost with the majority of the data in this paper. We asked one reviewer versed in drug pharmacokinetics to give us suggestions for improving readership and the other versed in the blood brain barrier.

We ask that you take their hard work to heart and move many of the details to supplemental and make the main data accessible to our readership in a more broad fashion. PLoS is committed to publishing all data whether negative or not. We hope that you take our suggestions seriously.**

**Response:** We have removed **10 pages** of methods and results text and placed them in the supplementary file. We have moved **one table** (new Table S5) to the supplementary file and combined two tables (Tables 11 and 12 **combined to form new Table 10**). We have focused (even more) the main results section to those where pentamidine and the selected pluronic F68 are described. We do not think that removing even more information to the supplemental file would enhance the manuscript further. We think it would just make it disjointed and more complicated to read.

**Reviewer 1.**

1. **The authors would benefit from more careful selection of what experimental designs, details, and results belong in the main body of the paper, and what could be most usefully put into the supporting information. I believe that this paper is too unfocused, and that the salient experimental results and interpretation should be severely streamlined.**

**Response:** We have removed **10 pages** of methods and results text and placed them in the supplementary file. We have moved **one table** (new Table S5) to the supplementary file and combined two tables (Tables 11 and 12 **combined to form new Table 10**). We have focused (even more) the main results section to those where pentamidine and the selected pluronic F68 are described.

We do not think that removing even more information to the supplemental file would enhance the manuscript further. We think it would just make it disjointed and more complicated to read.

**Some specific comments:**

**2. There are a good number of grammatical errors; the paper needs a careful re-read.**

**Response:** We have reread the manuscript and corrected grammatical errors. These changes have been highlighted.

**3.  Line 143 - how does the successful use of AmpB micelles indicate that pluronics "are also active agents with key biological functions"?**

**Response:** To explain this more clearly we have included additional information on lines 143 – 148.

‘Interestingly, F127-based amphotericin B-containing micelles have been shown to be highly effective in treating *Leishmania amazonensis*-infected BALB/c mice with results indicating that the empty micelles also exhibited antileishmanial activity (Mendonça *et al.*, 2016). Together these studies demonstrate that Pluronics have potential beyond the traditional role of simple micellar vessels for drug encapsulation and longer circulation, but are also active agents with key biological functions (Alakhova and Kabanov, 2014).

**4. Line 151. I think that if there's evidence that pluronics improve BBB penetration it would be good to reference that here.**

We have amended line 132 to more strongly state this:

**Response:** Line 132 states: Notably, Pluronic P85, P105, F68 and L61 have been shown to inhibit efflux transporters (including P-gp and MRP1-2) and have been shown to enhance drug passage across the BBB (Shaik, Pan and Elmquist, 2008) (Batrakova *et al.*, 1998)(Batrakova *et al.*, 1999) (Shaik, Giri and Elmquist, 2009) (Miller, Batrakova and Kabanov, 1999)(Rapoport, Marin and Timoshin, 2000)(Batrakova, 2003)(Fang *et al.*, 2013)(Wang *et al.*, 2012)(Venne *et al.*, 1996)(Huang *et al.*, 2008). We have also taken this opportunity to include another two references:

Batrakova E V, Li S, Miller DW, Kabanov A V. Pluronic P85 increases permeability of a broad spectrum of drugs in polarized BBMEC and Caco-2 cell monolayers. Pharm Res. 1999;16(9):1366–72.

Batrakova E V, Miller DW, Li S, Alakhov VY, Kabanov A V, Elmquist WF. Pluronic P85 enhances the delivery of digoxin to the brain: in vitro and in vivo studies. J Pharmacol Exp Ther [Internet]. 2001 Feb [cited 2016 Jan 18];296(2):551–7. Available from: http://www.ncbi.nlm.nih.gov/pubmed/11160643

**5. The Figure 1 content is very dense, the fonts are very small, and the color coding is not explained. In addition, the "SAR feedback loop" is never addressed in the paper -- how would the results feed back into the in silico and subsequent experiments? And I don't believe it's comment for the term "structure activity relationships" to be used referring to formulations...**

**Response:** We have now enlarged the figure presenting it in landscape view. We have now removed the colour coding as it is irrelevant in this manuscript. (It was originally used to identify staff involved in the specific activity).

We agree we do not use the exact words (i.e. structure activity relationship feedback loop), but we do mention an iterative approach was utilised in the figure 1 legend and on line 147 We also state on line 661:

‘As this was a milestone driven project an iterative, dynamic approach was utilized to select the lead formulation to take forward as quickly as possible in the screening cascade (Fig 1), hence not all Pluronic formulations were assessed with each of the methods’.

However, we have now modified the legend to use the exact words:

**‘Fig 1**. **NANOHAT project screening cascade. We used a structure activity relationship (SAR) feedback loop to further refine the selection of the lead formulations progressing through the screening cascade.** We screened approximately 30 pentamidine/Pluronic® formulations during this project using this rational, iterative approach. The three milestones were intended to ensure that the most appropriate formulations, on the basis of *in silico* and *in vitro* data, were taken forward to the *in vivo* pharmacokinetic studies and that the formulations with the greatest likelihood of success would be tested in the whole animal efficacy studies as outlined in the progression strategy’.

**6. Line 396 and following -- the last sentence of this paragraph is confusing....were only two healthy volunteers' blood used? If so, maybe just say this in the first sentence.**

**Response:** We have amended the first sentence line 382 to include this information and removed the last sentence.

7. **Line 471 - what is the difference between "permeability' and "accumulation" formats?**

We have now explained this in the text line 453:

‘Several *in vitro* permeability models in both accumulation (reflecting plasma into the endothelial cell) and permeability (reflecting plasma to brain interstitial fluid) formats were evaluated for this study’.

**8. In the animal studies, why were opposite sexes used in the two different mouse strains?**

**Response:** This was just availability of the mice who were otherwise to be culled.

9**. Table 3 - the last three entries show estimated Ki values. How were these estimated? And all three are estimated to be exactly 10 uM?**

**Response:** At stated in Table 3, it states that it was estimated from a single point screen. The single point was 10 microM.

**10. Table 4. Please consider whether four significant figures are appropriate here.**

**Response**: We have reduced it to three significant figures**.**

**11. Line 824 and following. I'm surprised to see that pluronics are so active in inhibiting insulin secretion...and/or that this wouldn't already have been a known phenomenon given the formulations' prevelance. IF it is know, this should be stated and referenced.**

**Response:** We are not aware of any previous publications stating this.

**12. Line 1061. I'm not sure that I saw that any MD simulations were performed, or how they were utilized (they don't seem to be mentioned anywhere else.**

**Response:**

Lines 757: The simulation results are shown in Figure 2 and Figure S6. We have now emphasised this in the accompanying results text.

To summarise **reviewer 2** comments:

1. **The question of the MDCK-MDR1 as a model of the BBB was questioned.**

**RESPONSE:** Line 454 has been changed from several *in vitro* BBB models…..to *in vitro* permeability models

The reviewer states this:

2. **The reason their MDR1-MDCK model failed to show this is because IT IS NOT A BBB MODEL IN THE FIRST PLACE.**

**RESPONSE**: This study explored the interaction of formulations with transporters expressed at the BBB using a variety of research tools. The transporter we were particularly interested in was human P-glycoprotein. We hoped the selection of the MDCK-MDR1 cell line would allow us to explore this transporter in more detail. In this way the MDCK-MDR1 is a model of the BBB.

3. **Future experimentation perfusion condition(s) should take into account physiologic SS flow and flow patterns based on normal (mouse?) heartbeat and flow rates. The data set obtained will certainly change, but whether the final outcome will be altered is something to think about.**

**RESPONSE**: Some of us has already explored this. Please see Sanderson et al., 2007.

We have also referred to this paper on lines 512 and 536.

4. **The review queries why the authors did not directly test the F68 formulations in trypanosome infection-based murine models of CNS HAT; i.e. Kaplan-Meier study or drug studies based on live IVM imaging using luciferase-labeled parasites.**

**RESPONSE**: This is to do with the severity of the animal procedures and therefore animal welfare and the early stage of development of the imaging using luciferace-labelled parasite at the time of this study The Infection based murine models of the CNS are classed as a more severe procedure than the *in situ* brain perfusion method which is performed under a terminal anaesthetic.

5. **The trypanosome toxicity studies: The trypanosome toxicity studies** shown brought up RED FLAGS. 10 nM pentamidine is trypanocidal. Yet **Table 9** that unformulated pentamidine at 015 nm is trypanocidal activity (kills 92.7%) of 0.15nM (100x lower than literature). Something wrong here??? Everyone else is wrong??? The lowest conc of pentamidine in **Table 9** is 5.7 x 10^-6^ µM, which is 5.7 pM. There should be a 0 uM drug column to have better idea of % tryps killed in present of Pluronic alone: Maybe their Tbb 427 strain was an ultrasensitive one? In reality, for future reference the data would have been more meaningful had the tests been done using *T b gambiense* parasites.

**RESPONSE:**

The IC_50_ for pentamidine against *Trypanosoma brucei brucei* strain 427 and *Tb gambience* has been reported as 1.8 to 26.1 nM and 14.7±4.7 nM respectively (Likeufack et al., 2006; Sykes et al., 2012). We have now included this in the methods Lines 183-185.

As stated in the text (line 846) we cannot use these pentamidine concentrations in Table 9 to determine a IC_50_, partly because of the high starting concentration used.

Please note *Tbb* is non-pathogenic to humans in contrast to *T b gambiense*. It is therefore safer to handle and so was selected for this study.

The effect of the various pluronics without pentamidine is already reported as an IC_50_ in Table 8.

Likeufack ACL, Brun R, Fomena A, Truc P. Comparison of the in vitro drug sensitivity of Trypanosoma brucei gambiense strains from West and Central Africa isolated in the periods 1960–1995 and 1999–2004. Acta Trop [Internet]. 2006 Nov;100(1–2):11–6. Available from: https://linkinghub.elsevier.com/retrieve/pii/S0001706X06001574

Sykes ML, Baell JB, Kaiser M, Chatelain E, Moawad SR, Ganame D, et al. Identification of Compounds with Anti-Proliferative Activity against Trypanosoma brucei brucei Strain 427 by a Whole Cell Viability Based HTS Campaign. Dumonteil E, editor. PLoS Negl Trop Dis [Internet]. 2012 Nov 29;6(11):e1896. Available from: https://dx.plos.org/10.1371/journal.pntd.0001896

6. **MIN6 -cell insulin secretion studies:** More to the point, the effect pentamidine and Pluronics on MIN6 -cell insulin secretion shown in Figure 3 were based only on 3 very high pentamidine concentrations 100 times, 1000 times and 10000 times the trypanocidal dose. From ***lines 832-836,*** it is clear that while all concentrations of P85 and P105 strongly suppressed of insulin secretion from MIN6 β-cells. However, compared to free pentamidine the F68 formulations only induced insulin secretion suppression at concentrations ≥0.1% w/v. Not much is said about the 20% increase in insulin secretion at the 1 μM free/F68 pentamidine dose!!! This reviewer suggests that this study should be redone using sub to high nM of low μM range as there may be some hidden message (protective mechanism?) here.

**RESPONSE:**

This is an interesting observation and was one of the reasons for selecting F68 as our lead formulation for further *in vivo* evaluation as mentioned here Lines 1090-1091. However, the CNS evaluations did not suggest that we could increase the delivery of 15.7 nM radiolabelled pentamidine into the brain with F68 so we chose to close the study rather than further explore the effect of lower concentration of pentamidine on insulin secretion in MIN6-cell line.

7. **CNS side effects: *Lines 193-197*** states ‘…A CNS side effect panel was custom designed and binding assays performed by Perkin-Elmer Science Discovery Systems…Testing was performed at a single concentration of 1 μM (100-times the trypanocidal concentration), with follow up concentration-response curves in any assay where there was greater than 70% inhibition to determine an inhibition constant (Ki)***.***  And yet ***Lines 654-657*** states that ‘...A wide ligand profiling screen was carried out against 40 CNS targets (Perkin Elmer customised CNS screening; listed in **Table S1**), testing at a single concentration of 10 μM (1000-times the trypanocidal concentration), with follow up concentration-response curves in any assay where there was greater than 70% inhibition.’ First of all, there is no real explanation on how Perkin-Elmer did the actual assays and the context for the basis of the 40 CNS targets. Clear this was a justification for a quick CNS toxicity assay rather than doing the tests properly. The correct way would have been to serially dilute the formulations starting from sub-nM range based on pentamidine content upwards, certainly no higher than 1 μM.

**RESPONSE:**

We have now included more details of the standard radioligand assay by enhancing Table S1 and legend. In this assay pentamidine is only considered active at a binding site if it shows an inhibition of 50% or greater. Hence this initial rapid screen identified binding sites of interest for more detailed assessment of relevant interactions as shown in Table 3.
